# Effects of Tai Chi and Walking Exercise on Emotional Face Recognition in Elderly People: An ERP Study

**DOI:** 10.3390/healthcare10081486

**Published:** 2022-08-07

**Authors:** Xiaofei Zhang, Jie Bao, Haoping Yang, Zonghao Zhang, Deming Shu, Li Luo

**Affiliations:** 1 School of Physical Education and Sports Science, Soochow University, Suzhou 215021, China; 2 School of Education, Soochow University, Suzhou 215023, China

**Keywords:** walking, tai chi exercise, emotional face, recognition, memory, aging, event-related potential

## Abstract

Both tai chi and walking can improve the physical health of the elderly, but the effect on the emotional cognitive function of the elderly is unclear. To investigate the effect of long-term walking and tai chi exercise on the emotional cognitive function of the elderly, 63 subjects were recruited in this study according to age and exercise habits, including 16 in the youth control group, 15 in the elderly non-exercise control group, 17 in the elderly walking group, and 18 in the elderly tai chi group. The “learning–test paradigm” of emotional faces was used to measure the subjects’ ability to recognize and remember emotional (negative and neutral) faces. Behavioral and EEG data were recorded during the learning and testing phases. The results showed that there is aging in emotional cognition in older adults compared with younger adults. Long-term walking and tai chi exercise can delay the deterioration of emotional cognitive function in older adults to some extent. Both walking and tai chi exercise can delay the decline in aging-related emotional face recognition function to some extent. Walking exercise can delay the decline in aging-related emotional face memory function to some extent.

## 1. Introduction

Population aging has become a worldwide social phenomenon [1].With aging, there is commonly a decline in learning and memory ability, reducing the quality of life and increasing the economic burden of society [2].Therefore, how to delay the degeneration of brain function caused by aging is an important issue of concern.

People are paying more attention to mental health, and emotions are one of the important components of mental health [3]. In 1972, S. Schachter and J.E. Singer proposed the cognitive theory of emotion, which holds that the factors affecting emotion are cognition, physiology, and environment, and the core of the emotional arousal model is cognition [4]. Emotion and cognition are separate mental processes with independent changes and patterns, but at the same time, they are also closely related. Emotion has a certain organizing effect on cognition, and positive emotions can positively contribute to cognitive activities [5], which largely depends on the intensity of emotion.

Emotional experience refers to an emotional state that a person feels or is aware of subjectively [6]. Individuals at different stages of development differ in their positive and negative emotional experiences, with negative emotions having a greater impact on the human brain, mind, and body than positive emotions [7]. Many studies have found that the frequency of positive emotional experiences and subjective well-being increases gradually during aging, while the frequency of negative emotional experiences decreases [8,9]. Older adults benefit from the same memory-enhancing effects of emotions as younger adults, but emotional regulation in older adults may often take the form of “suppression” of negative memories rather than promotion of positive memories [10].

The phenomenon of cognitive and neural plasticity suggests that training can lead to improved or even reversed cognitive function in older adults and has an impact on improving brain volume and brain function [11,12]. In physical activity, emotions influence and accompany the entire process of motor skill learning and movement completion [13]. Previous studies have shown that physical activity can have a positive impact on all aspects of the body and mind of older people [14], enhance disease resistance [15,16], and help them to improve their quality of life [17]. Physical activity can improve a person’s emotional state [18], but not all physical activities have this effect.

Exercise and cognitive training can improve brain-related health in older adults [19]. Tai chi is a Chinese intangible cultural heritage that improves physical and mental health and is as easy to learn, safe, convenient, and popular as walking. Numerous studies have confirmed that walking and tai chi exercise can promote older people’s physical and mental health, including improvement in cognitive function [20,21]; however, the underlying mechanisms are still far from clear. Tai chi and brisk walking offer some of the benefits of exercise for cognitive function in older adults, and tai chi is more beneficial than brisk walking for cognitive function (executive and non-executive) in older adults [22].

Face are special kinds of visual stimulus that contain a rich content. Face recognition research is an important and active part of the research field of psychology and cognitive science. Facial expressions are an important way of expressing emotion, reflecting the individual’s psychological and physiological state and interaction with the external environment [23]. Therefore, the ability to accurately distinguish and memorize faces, judge the emotions and intentions of others from their facial expressions, and respond appropriately is related to the success or failure of individuals’ social interactions and communication [24]. Many studies have shown that older adults have difficulties in recognizing facial anger, sadness, and fear, which may lead to a number of social dysfunctions, including impaired social competence, decreased social interest, and inappropriate social behavior, thus affecting their quality of life [25]. Therefore, it is meaningful to probe into the characteristics and patterns of emotional face recognition and memory and the factors influencing them in older adults. During aging, degeneration of the brain will cause cognition decline [26]. The ability to recognize and remember human faces gradually decreases with age in the elderly.

This study adopts the emotional faces learn–test paradigm and examines the effect of long-term walking and tai chi exercise on the emotional face recognition and memory of the elderly, the influence of emotional valence on the elderly’s emotional face recognition and memory, and the possible neural mechanisms to help establish a theoretical and experimental basis for healthy aging.

## 2. Method

### 2.1. Participants

Based on the number of subjects reported in related studies [27] and G*Power 3.1.7, the sample size was calculated. Repeated measurement ANOVA was used as a statistical test for Experiment 1, the parameter effect size f was set to 0.25, the probability of class I error α err prob was 0.05, the test efficacy power (1 − β err prob) was 0.8, and the sample size obtained was 52.

In order to recruit subjects, relevant information was posted on community and online platforms. Sixty-six participants (34 men, 32 women) were actually recruited based on age and physical activity history and divided into one of four gender-balanced groups: an elderly tai chi group (18 in total), elderly walking group (17 in total), elderly control group (15 in total), and young control group (16 in total). Young people between the ages of 20 and 30 and older people between the ages of 60 and 80 were included in the study. After reviewing the selection criteria of the long-term physical exercise literature, the subjects in the walking and tai chi exercise groups were included based on the following criteria: at least 5 years of exercise, more than 1 h per day of exercise, and more than 5 days per week of exercise. Criteria for non-exercise were as follows: able to live and study normally, habitually sedentary, occasional walking, exercise time less than 20 min per day, and exercise frequency less than 2 days per week. Subjects in each group were equally divided between men and women. The demographic information and physical activity characteristics of the participants are presented in Table 1. All reported no neurological disorders, cardiovascular disease, or use of any medications affecting central nervous system function, and had normal or corrected-to-normal vision. Subjects had no serious physical illness and no history of smoking or drinking. None of the subjects had been volunteers previously. All of the subjects provided written informed consent prior to participation, and the study was approved by the Ethics Committee of Soochow University.

### 2.2. Stimulus Materials

The stimulus materials consisted of 232 color portraits of unknown faces, all with hair removed and without glasses, from which 64 face pictures were selected as stimulus materials for the learning phase (32 negative pictures and 32 neutral pictures) and 168 faces were selected as novel faces for the testing phase (84 negative pictures and 84 neutral pictures); all were taken from the Chinese Affective Face Picture System (CAFPS) by Beijing Normal University. The selection of faces was balanced by gender and emotional valence, and the response keys were balanced between subjects. The valence and arousal of the face materials were rated, and all negative and neutral stimulus pictures showed significant differences in valence and arousal (Table 2 and Table 3) [28]. All of the images were edited to a uniform format (10.4 × 8 cm), had the same grayscale and luminance background (8-bit color scale), and were framed in a 350 × 270 pixel area. The procedure consisted of a learning phase and a test phase, 1 h apart. During the test, emotional face pictures were presented on a gray background, and stimulus pictures were superimposed on a dark gray background on a 17-inch CRT computer screen placed 1 m in front of the subject, using E-Prime 2.0 stimulus presentation software, which recorded the subject’s correctness and response time.

### 2.3. Procedure and Design

Subjects needed to be well rested before the experiment to ensure that they were in good condition, wash and blow dry their hair before the test to ensure that their scalp was dry and free of grease, and wear electrode caps to ensure that information could be collected properly. The subjects were informed in advance of the experiment that they would see some neutral and negative face pictures. The subjects were first given a practice session to familiarize themselves with the entire experimental procedure. The formal experimental procedure for each subject consisted of two parts: the learning phase and the testing phase. The two were separated by 1 h.

(1) Learning phase: Four blocks of 64 pictures each were presented randomly, for a total of 256 trials. Subjects were presented with a green “+” for 600 ms, a face picture for 4000 ms, and a white “+” for 2000 ms. Subjects were required to judge the face as quickly and accurately as possible using their left and right index fingers, pressing the F key on the keyboard for a neutral expression and the J key for a negative expression, and to try to remember the faces that appeared to complete the later recognition task. The reaction keys were balanced between subjects.

(2) Test phase: This phase consisted of blocks, each round consisting of 64 target faces, 56 novel faces (28 negative, 28 neutral), and 8 filler face recurrences, presented as a green “+” for 600 ms, a face picture for 3000 ms, and a white “+” for 2000 ms. No post-analysis was performed.

**Figure 1:**
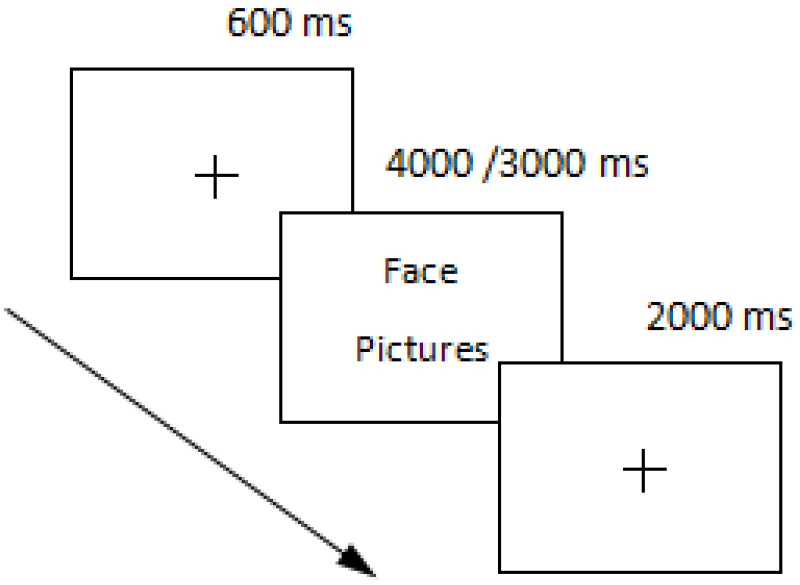
Experimental design.

Subjects were required to judge the faces as quickly and accurately as possible using their left and right index fingers and determine whether the faces were new or old, pressing the F key on the keyboard for new faces and the J key for old faces. The reaction keys were balanced between subjects. To record the responses more accurately, the subjects needed to keep their left and right index fingers placed at the corresponding position on the keyboard, during which the subject was encouraged to keep their body as still as possible and avoid physical movements such as shaking the legs or yawning frequently. There was a break prompt during the test, and the subject could move his or her body slightly during the break. The participants were reminded to follow the instructions and keep quiet at the beginning of the experiment and were reminded of the time required for the experiment, which lasted approximately 1.5 h (Figure 1).

### 2.4. Electrophysiological Recording

**Figure 2:**
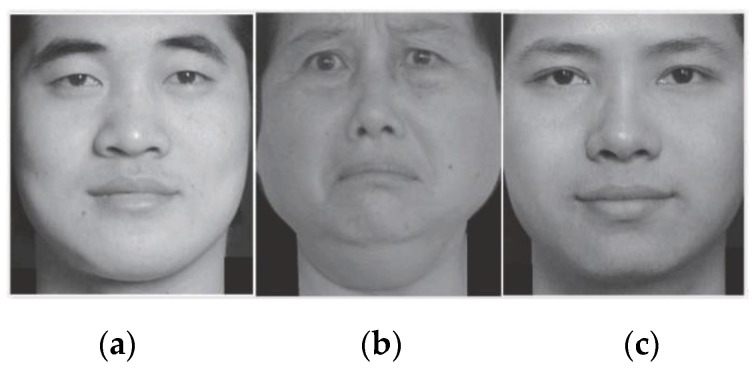
Representative photos of the experiment. (**a**) Neutral face, (**b**) negative face, and (**c**) neutral face.

The experiments were conducted in a quiet and soundproof room at a room temperature of about 25 °C (Figure 2). A Brain Products ERP EEG recording and analysis system was used, and the EEG data were collected from a 32-electrode cap extended from the common international 10–20 system. The horizontal electrooculography (HEOG) and vertical electrooculography (VEOG) were recorded simultaneously using electrode caps, averaged over TP9 and TP10 as references and AFz ground electrodes, respectively. The filtered bandpass was 0.05 to 100 Hz, the A/D sampling frequency was 500 Hz, and the impedances on all electrodes were measured and confirmed to be less than 5 kΩ both before and after testing. The offline analysis of the EEG was analyzed using Analyzer 2.0 software, and the EEG was corrected by an algorithm described by Gratton and Coles (1989). The segmentation epoch was 200 ms before to 1400 ms after stimulus onset. Only correct responses were analyzed.

### 2.5. Statistical Analysis

All of the data were processed using SPSS 18.0 software, and ERP data were normally distributed according to a Kolmogorov–Smirnov test (K–S test). All of the dependent variables were subjected to repeated measures ANOVA with a 5% error level, and *p*-values were corrected using the Greenhouse–Geisser method. Post hoc comparisons were made using independent samples *t*-tests, and *p* < 0.05 was considered to be statistically significant.

#### 2.5.1. Behavioral Data Statistics and Analysis

Repeated measures ANOVA was performed on the correctness and reaction time of the face valence recognition of subjects in the learning phase using group (youth control group, elderly control group, elderly walking group, and elderly tai chi group) as a between-subjects variable and face valence as a within-subject variable; group (youth control group, elderly control group, elderly walking group, and elderly tai chi group) was used as a between-subjects variable and face (old or new) as a within-subject variable. Repeated measures ANOVAs were conducted on the correctness and reaction times of subjects’ old and new face memories during the testing phase. Only the reaction times of correct responses were analyzed.

#### 2.5.2. ERP Data Statistics and Analysis

A total of six electrodes in the frontal (F3, Fz, F4) and parietal (P3, Pz, P4) regions were selected for analysis, and the wave amplitudes evoked by the target stimuli were measured. According to pre-experiments and the observation of ERP waveforms, the differences in the peak latencies and peaks of N1, P1, N170, N3, and P3 in the frontal and parietal regions of the hemispheres (left, middle, and right) and potencies (negative and neutral) were analyzed for different groups of subjects in the learning phase; in the testing phase, the differences in the average amplitudes of ERP evoked in the frontal and parietal regions of the hemispheres (left, middle, and right) were analyzed for different groups of subjects in the 380–500 ms and 500–700 ms ranges. In the test phase, we analyzed the differences in the mean ERP amplitudes between different groups of subjects in the hemispheres (left, middle, and right), old and new faces, and face potencies (negative, neutral) [29].

## 3. Results

### 3.1. Behavioral Data

#### 3.1.1. Study Session

**Figure 3:**
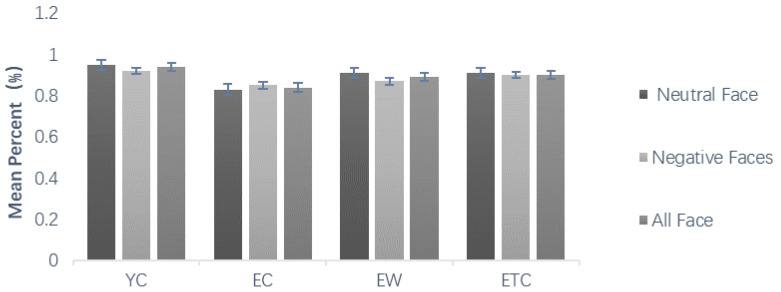
Comparison of mean percent accuracy associated with the learning task (%).

Repeated measures ANOVAs on the correctness of emotional face recognition during the learning phase, using group (youth control YC, elderly control EC, elderly walking EW, and elderly tai chi ETC) as the between-subjects variable and face emotional valence as the within-subject variable, showed that the within-subject difference in emotional valence was not significant, *F*(1, 59) = 1.210, *p* = 0.276, *η_P_*^2^ = 0.020. The post hoc multiple comparison results revealed that the older control group was significantly less accurate than the younger control group at identifying the emotional faces (*p* < 0.001). There was no significant difference between the elderly walking group or the elderly tai chi group and the youth control group (*p* = 0.099, *p* = 0.511). The correct rate of the elderly walking group and the elderly tai chi group was significantly greater than that of the elderly control group (*p* < 0.05, *p* < 0.05), and there was no significant difference between the elderly walking group and the elderly tai chi group (*p* = 0.955) (Figure 3).

**Figure 4:**
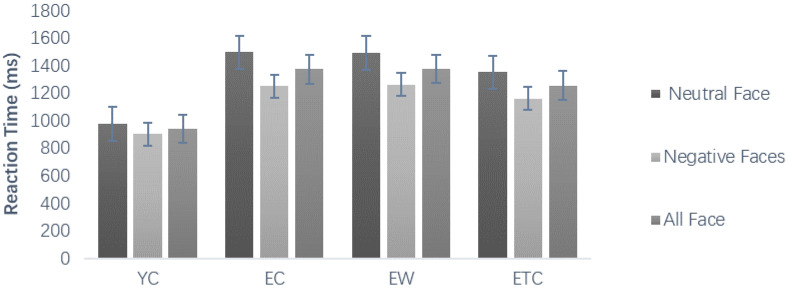
Comparison of reaction time accuracy associated with learning task (ms).

Repeated-measures ANOVAs were conducted on the reaction times of correct responses during the learning phase using group (youth control, elderly control, elderly walking, and elderly tai chi groups) as the between-subjects variable and face valence as the within-subject variable, as shown in Figure 4. The within-subject effect of reaction time for face valence was significant, *F*(1, 59) = 30.603, *p* < 0.001, *η_P_*^2^ = 0.342. The interaction between emotional valence and group was not significant. The between-subjects difference was significant, *F*(3, 59) = 6.632, *p* = 0.001, *η_P_*^2^ = 0.252, and further pairwise comparisons revealed that the response time of the elderly control group was significantly longer than that of the youth control group (*p* < 0.01). The differences between the elderly walking group and the elderly control group, between the elderly tai chi group and the elderly control group, and between the elderly walking group and the elderly tai chi group were not significant (*p* > 0.05).

#### 3.1.2. Test Session

**Figure 5:**
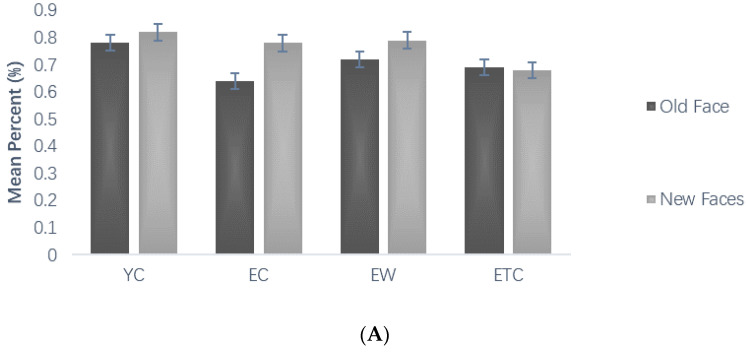

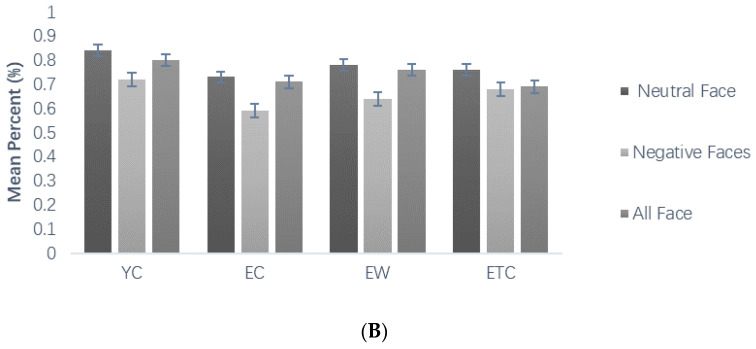
Comparison of mean percent accuracy associated with the memory task (%). (**A**) The average mean percent of different groups in old and new faces, (**B**) the average mean percent of different groups in neutral, negative and all faces.

Repeated measures ANOVAs on correct face discrimination rates during the testing phase, using group (youth control, elderly control, elderly walking, and elderly tai chi) as between-subject variables and new/old faces and face emotional valence as within-subject variables, showed significant within-subject main effects for new and old faces, *F*(1, 59) = 6.510, *p* < 0.05, *η_P_*^2^ = 0.099, and old faces were significantly more correct than new faces, i.e., subjects tended to mistake new faces for old faces (Figure 5A); the interaction between new and old and group was not significant, *F*(3, 59) = 0.777, *p* = 0.511, *η_P_*^2^ = 0.038, and the main effect of face emotional valence was significant, *F*(1, 59) = 38.049, *p* < 0.001, *η_P_*^2^ = 0.392. The post hoc multiple comparison results showed that, in terms of correct discrimination of all faces, the elderly control group and the elderly Tai chi group were significantly smaller than the youth control group (*p* < 0.01). The control group (*p* < 0.05, *p* < 0.01) and the youth group were able to identify the emotions in the experimental material more accurately than the older Tai chi and walking groups (*p* < 0.05). The performance of the elderly Tai chi group was not significantly different from that of the elderly control group (*p* = 0.858), while that of the elderly tai chi group was significantly poorer than that of the elderly walking group (*p* < 0.05, Figure 5B).

**Figure 6:**
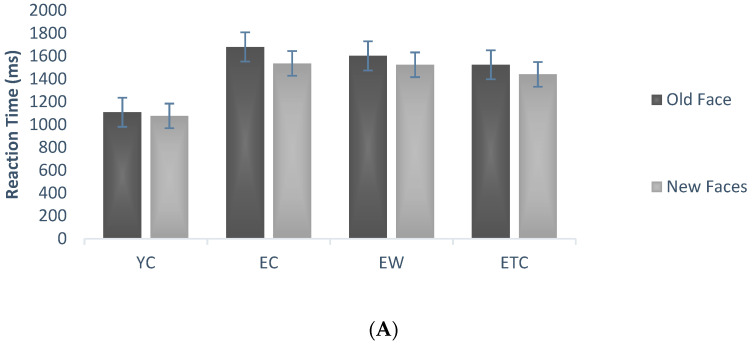

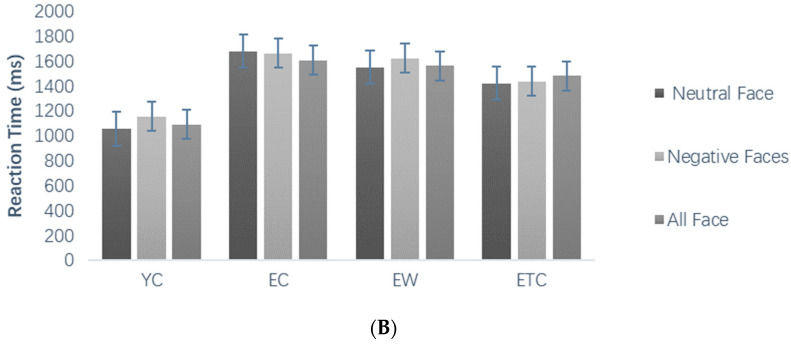
Comparison of reaction time associated with memory task (ms). (**A**) The average reaction time of different groups in old and new faces, (**B**) the average reaction time of different groups in neutral, negative and all faces.

Repeated measures ANOVAs were conducted on the reaction time to correct face discrimination during the testing phase using group (youth control, elderly control, elderly walking, and elderly tai chi) as the between-subjects variable and face old and new and face emotional valence as the within-subject variables, showing that the main effect of old/new faces was significant, *F*(1, 59) = 4.682, *p* < 0.05, *η_P_*^2^ = 0.074, and the subjects reacted significantly less often to old faces than to new faces (Figure 6A). The interaction between new and old faces and group was not significant, *F*(1, 59) = 0.185, *p* = 0.906, *η_P_*^2^ = 0.009. The main effect of face emotional valence was significant; subjects reacted significantly more often to neutral faces than to negative faces, *F*(1, 59) = 29.472, *p* < 0.001, *η_P_*^2^ = 0.333. The interaction of face emotional valence and group was not significant, *F*(3, 59) = 0.874, *p* = 0.460, *η_P_*^2^ = 0.043. The difference between subjects by group was significant, *F*(3, 59) = 7.971, *p* < 0.001, *η_P_*^2^ = 0.288. Post hoc multiple comparison results showed that the response times for correct discrimination in the older control, older walking, and older tai chi groups were significantly longer than those of the youth control group (*p* < 0.01, *p* < 0.01, *p* < 0.01), and there was no significant difference between the elderly walking and elderly tai chi groups compared with the elderly control group (*p* = 1.000, *p* = 0.750, Figure 6B).

### 3.2. ERP Data

#### 3.2.1. ERP Components of Emotional Face Recognition

**Figure 7:**
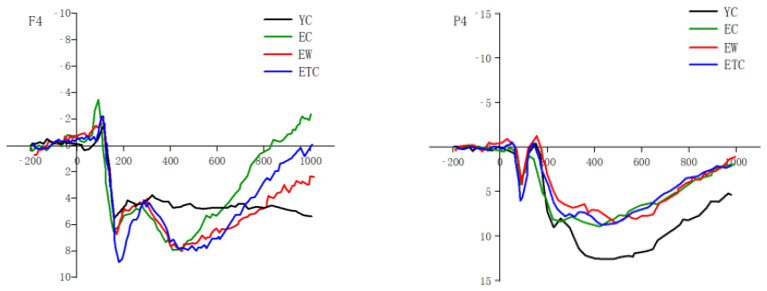
ERPs of different groups of subjects identifying negative emotional expression (left: F4, right: P4).

By conducting peak detection of N1, P1, N170, N3, and P3 in the frontal and parietal regions for different groups of subjects, the time windows of detection were as follows: N1: 60 ms to 140 ms; P1: 60 ms to 140 ms; N170: 130 ms to 210 ms; N3: 230 ms to 290 ms; P3: 400 ms to 600 ms. The results showed that more obvious N1, P1, N170, N3, and P3 were induced (Figure 7). Peak latencies and peaks were derived after detection, and subsequent repeated measures ANOVAs with group as a between-subjects variable and hemispheres (left, center, right) and emotional valence (negative, neutral) as within-subject variables were performed for each waveform in the frontal (F3, Fz, F4) and parietal (P3, Pz, P4) regions, respectively.

The results of the peak and latency analysis of N1 showed that the peak and peak latency of N1 in the frontal region were not significant in either intra- or inter-subject effects. In the peak of the parietal region, the intra-subject main effect and inter-subject effect were not significant. The intra-subject hemisphere effect of peak latency was significant, *F*(2, 118) = 3.817, *p* < 0.05, and pairwise comparisons revealed that the middle latency was significantly smaller than that of the left and right hemispheres. The inter-subject effect of peak latency was not significant.

The results of the peak and latency analysis of P1 showed that the within-subject main effect and between-subject effect were not significant for the peak in the frontal area P1. The hemispheric main effect was significant for the peak in parietal area P1, *F*(2, 118) = 10.938, *p* < 0.001, *η_P_*^2^ = 0.156, and pairwise comparisons revealed that the middle wave amplitude was significantly larger than that of the left and right hemispheres. The interaction between the peak and the group was significant, *F*(6, 118) = 2.979, *p* < 0.05, *η_P_*^2^ = 0.132, and none of the between-subject effects for peak were significant. In the peak latency of frontal area P1, both intra- and inter-subject differences were not significant. In the peak latency of parietal area P1, the intra-subject hemispheric difference was significant, *F*(2, 118) = 4.804, *p* < 0.05, *η_P_*^2^ = 0.07. Further pairwise comparisons revealed that the central part was significantly larger than the left (*p* < 0.05) and right (*p* < 0.01), and inter-subject differences were not significant.

**Figure 8:**
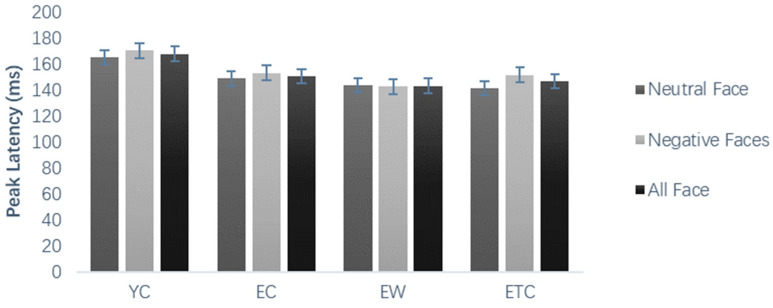
N170 frontal area peak latency (ms) in different groups of subjects’ learning stages.

The results of the peak and latency analysis of N170 and N170 peaks in the frontal region did not show significant within- or between-subject differences. For the N170 peak in the parietal region, the hemisphere within-subject effect was significant, *F*(2, 118) = 21.686, *p* < 0.0001, *η_p_*^2^ = 0.269; the central wave amplitude was significantly greater than that of the left and right hemispheres; and the hemisphere and group interaction was significant, *p* < 0.05. The peak latency of N170 in the frontal region, as shown in Figure 8, was not significant for the main effect of face valence, with significant between-subject differences, *p* < 0.05, and further pairwise comparisons revealed that the peak latency of the elderly control group was significantly smaller than that of the young control group (*p* < 0.05). The peak latency of N170 in the parietal region did not show significant within-subject or between-subject differences.

**Figure 9:**
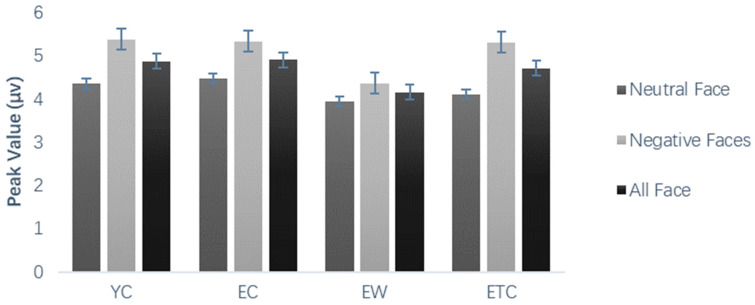
Peak (µv) of the N3 top region in different groups of subjects’ learning stages.

As shown in Figure 9, the results of peak and peak latency analysis for N3 and N3 frontal area peaks showed significant within-subject variation for face valence, *F*(1, 59) = 11.427, *p* = 0.001, *η_p_*^2^ = 0.162. The negative-face-induced wave amplitude was significantly greater than the neutral-face-induced one, with non-significant between-subject variation. The N3 parietal area peak showed significant within-subject variation for hemisphere, *F*(2, 118) = 12.487, *p* < 0.001. Pairwise comparisons showed that the middle was significantly greater than the left hemispheres (*p* < 0.001) and right hemispheres (*p* < 0.001). There were significant interactions with face valence within subjects (*p* < 0.05). N3 peak latencies in the frontal and parietal regions were not significantly different within or between subjects.

**Figure 10:**
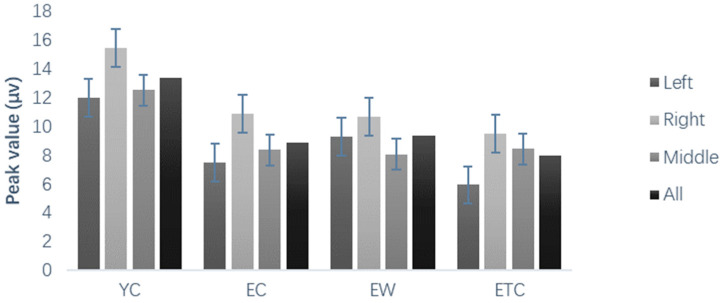
Peak (µv) of the P3 top region in different groups of subjects’ learning stages.

The results of the peak and peak latency analysis of P3 and P3 frontal area peaks showed that both within- and between-subject differences were not significant. As shown in Figure 10, the P3 parietal area peak showed significant within-subject differences in the hemispheres, F = 23.667, *p* < 0.001, *η_p_*^2^ = 0.286, with the center significantly greater than that of the left and right hemispheres. The main effect of face valence was significant, *p* < 0.001; the between-group main effect was significant between subjects. For the P3 frontal area peak latency, both intra- and inter-subject effects were not significant. For the P3 parietal area peak latency, the hemisphere effect was significant, *F*(2, 118) = 3.928, *p* < 0.05, *K* = 0.062, and the parietal central latency was significantly greater than the right latency (*p* < 0.01).

#### 3.2.2. Effects of Old and New Emotional Faces

**Figure 11:**
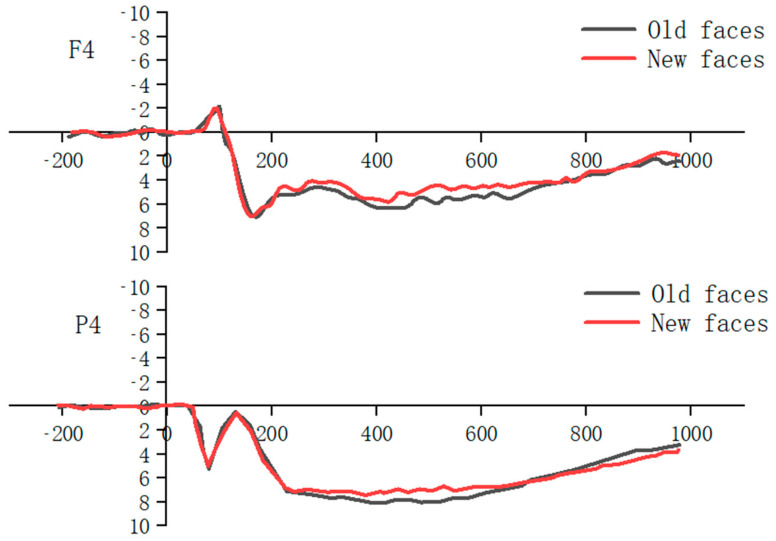
Waveform plots of old and new effects for all subjects (left: F4, right: P4).

A repeated measures ANOVA was conducted by comparing the mean wave amplitudes of ERPs evoked in the frontal and parietal regions at 380–500 ms and 500–700 ms, respectively, in different groups (youth control, elderly control, elderly walking, and elderly tai chi groups) of subjects in terms of hemispheres (left middle right) × old and new (new face, old face) × emotional valence (negative, neutral) (Figure 11).

The results of the repeated-measures ANOVA comparing the mean wave amplitudes in the 380–500 ms and 500–700 ms frontal areas with hemispheres (left, center, right) × old and new (new face, old face) × emotional valence (negative, neutral) as within-group factors and group as a between-group factor showed that the ERP old and new effects on the mean wave amplitudes of ERPs induced in the 380–500 ms frontal area for subjects in different groups were significant, *F*(1, 59) = 5.034, *p* < 0.05, *η_P_*^2^ = 0.079, with the mean wave amplitude of old faces being significantly greater than that of new faces. The main effect of emotional valence was significant, *F*(1, 59) = 9.115, *p* < 0.01, *η_P_*^2^ = 0.134, and the wave amplitude induced by negative faces was significantly greater than that of neutral faces. The mean ERP wave amplitude evoked in the frontal region from 500 to 700 ms was not significantly different between subjects in different groups (*p* > 0.05).

**Figure 12:**
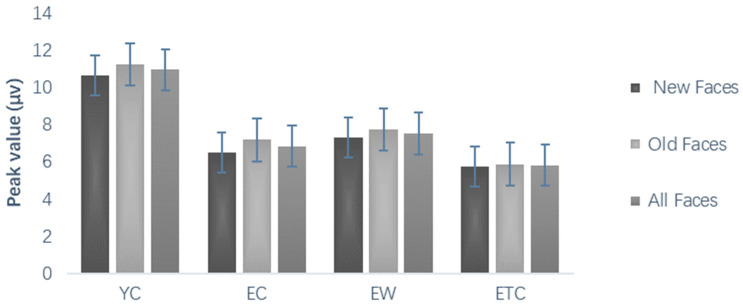
Effects of old and new faces in the top region of 380–500 ms for different groups of subjects (µv).

**Figure 13:**
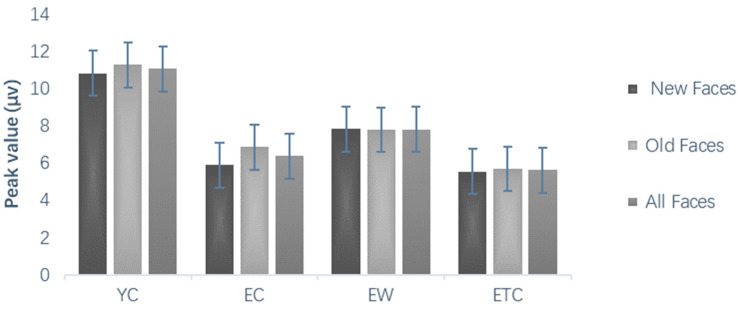
Effects of old and new faces in the top region of 500–700 ms for different groups of subjects (µv).

The results of the comparison of the differences in mean wave amplitude in the 380–500 ms and 500–700 ms parietal regions were analyzed by repeated measures ANOVA using hemispheres (left, center, right) × old and new (new face, old face) × emotional valence (negative, neutral) as within-group factors and group as a between-group factor. As shown in Figure 12, the mean ERP wave amplitude evoked in subjects in different groups in the parietal region at 380–500 ms showed that the hemispheric main effect was significant, *p* < 0.001; the old and new effect was significant, *p* < 0.01; and the face valence main effect was significant. There were significant differences between subjects, *F*(3, 59) = 4.538, *p* < 0.01, *η_P_*^2^ = 0.187. The mean wave amplitude of the elderly control group was significantly smaller than that of the youth control group (*p* < 0.01). The mean wave amplitude of the elderly walking group was larger than that of the elderly control group, but the difference was not significant (*p* > 0.05). The mean wave amplitude of the elderly tai chi group was not significantly different from that of the elderly control group (*p* > 0.05). The mean wave amplitude of the elderly walking group was greater than that of the elderly tai chi group, but the difference was not significant (*p* > 0.05). As shown in Figure 13, the mean wave amplitude of ERP evoked in the top region of 500–700 ms for subjects in different groups differed significantly in the hemispheric main effect, *p* < 0.001, which was significantly greater in the center than in the left and right hemispheres, with significantly greater old and new effects, *p* < 0.05, and significantly greater wave amplitude evoked by the old faces than by the new faces. There were significant differences between subjects, *F*(3, 59) = 4.740, *p* < 0.01, *η_p_*^2^ = 0.194. Pairwise comparisons revealed that the mean ERP wave amplitude evoked in the top region at 500–700 ms was significantly smaller in the elderly control group than in the young control group (*p* < 0.01). The mean wave amplitude of the elderly walking group was larger than that of the elderly control group, but the difference was not significant (*p* > 0.05). The mean wave amplitude of the elderly tai chi group was not significantly different from that of the elderly control group (*p* > 0.05). The mean wave amplitude of the elderly walking group was greater than that of the elderly tai chi group, but the difference was not significant (*p* > 0.05).

## 4. Discussion

### 4.1. Effects of Walking and Tai Chi Exercise on the Emotional Cognition of Older Adults

#### 4.1.1. Effects of Walking and Tai Chi Exercise on Emotional Face Recognition of Older Adults

In terms of the correct rate of the learning phase, there were significant differences between groups, and pairwise comparisons revealed that the correct rate of face recognition in the elderly control group was significantly lower than that in the young control group (*p* < 0.01); the correct rate in the elderly walking group was higher than that in the elderly control group (*p* < 0.05); the correct rate in the elderly tai chi group was significantly higher than that in the elderly control group (*p* < 0.01); and the difference between the elderly walking group and the elderly tai chi group was not significant. In terms of the reaction time of the learning phase, the reaction time of the elderly control group was significantly longer than that of the youth control group (*p* < 0.01); the differences in reaction time between the elderly walking group and the elderly control group, between the elderly tai chi group and the elderly control group, and between the elderly walking group and the elderly tai chi group were not significant (*p* > 0.05). Comprehensive analysis showed that both the elderly walking group and the elderly tai chi group had better recognition of emotional faces than the elderly control group, which indicates that long-term walking and tai chi exercise can improve the emotional face recognition ability of the elderly to some extent. Previous studies have shown that walking exercise can distract the mind and relieve mental stress, and one study using the Simple Health Survey Scale (SF-36) found that short-term walking exercise significantly improved anxiety levels in patients with menopausal anxiety disorder. Furthermore, a study of the P300 component of tai chi exercise found that tai chi, a moderate-intensity aerobic exercise, temporarily improved cognitive performance. In a study of older adults, Hatta et al. found that the response time of the physical exercise group was shorter than that of the non-exercise group and the P300 wave amplitude of the exercise group was significantly larger at the Pz point than at the Cz and Fz points, while the P300 wave amplitude of the non-exercise group was significantly larger at the Cz and Fz points. Fz and Pz points in the wave amplitude were the same in the non-exercise group, and the results suggested that regular moderate-intensity exercise in older adults has a positive effect not only on response processes, but also on cognitive processes [30]. The present study is consistent with previous studies.

#### 4.1.2. Effects of Walking and Tai Chi Exercise on Emotional Face Memory Behavior in Older Adults

In terms of the correct rate of the test phase, the elderly control group had a significantly lower correct rate of old and new face memory than the young control group (*p* < 0.05), indicating a degradation of face memory with age. The correct rate of the elderly walking group was greater than that of the elderly control group (*p* < 0.05), and there was no significant difference between the elderly tai chi group and the elderly control group (*p* > 0.05). The correct rate of the elderly walking group for old and new face memory was significantly greater than that of the elderly tai chi group (*p* < 0.05). In terms of test phase reaction time, the elderly control group had a significantly longer reaction time than the young control group and, between the elderly groups, the elderly tai chi group had a shorter mean reaction time relative to the elderly walking group and the elderly control group. Aging is a gradual and dynamic process of “gain” and “loss”. This process leads to a gradual decline in perceptual and cognitive abilities, especially in situational memory, and older adults have difficulty in performing “recall” tasks that require conscious information retrieval [31]. Some studies have shown that the volume of human brain tissue and cerebral blood flow tend to decline during aging, and older adults show declines in a variety of cognitive functions [32], but the results of different studies are inconsistent in terms of age differences in emotionally regulated memory, with some studies finding that older adults show declines in general memory performance compared with younger adults, but emotional memory is more intact [33,34]. The results of some studies showed no difference between older and younger people in the evaluation of positive pictures. However, older adults rated negative versus neutral pictures significantly more correctly than younger adults [35]. Combining the correctness and response time in the test phase, this study found that, in emotional face memory, there was a significant degradation of emotional face memory in the elderly control group relative to the young control group; long-term walking exercise could delay the degradation of emotional face memory function to some extent, and tai chi exercise had no significant effect.

### 4.2. Differences in ERP Components and Old and New Effects of Walking and Tai Chi Exercise on Emotional Cognition in Older Adults

#### 4.2.1. Effects of Walking and Tai Chi Exercise on ERP Components of Emotional Face Recognition in Older Adults

Previous studies have shown that faces’ emotional valence modulates the ERP components of the cognitive appraisal and perceptual stages of face emotion processing, with early anterior N1 and posterior P1 being biased toward the processing of negative expressions, negative expressions evoking larger waves than positive and neutral expressions, N170 waves evoked in the VPP and short interval conditions distinguishing between emotional and non-emotional faces, and N3 and P3 waves in the short interval clearly distinguishing between emotional and non-emotional faces. The N3 and P3 amplitudes at short intervals can clearly distinguish among negative, positive, and neutral expressions. Similar to previous ERP studies of emotional faces, emotional faces in the present study evoked more pronounced N1, P1, N170, N3, and P3 components, all of which showed significant main effects on emotional valence as well as between-subject differences across age groups. Many studies have shown that components such as N170 and P300, which are triggered by specific stimulus paradigms, are associated with the cognitive processing of emotions. Analysis of the changes in the amplitude and distribution of ERP components triggered by different emotional components can reveal the neural mechanisms of emotion and emotion regulation.

N170 is a negative component distributed on both sides of the occipitotemporal scalp, with a peak of about 170 ms. Recent studies have shown that facial expressions, race, familiarity, and gender do not affect N170, which suggests that face recognition is specific [36]. In the present study, the N170 peak latency in the frontal area differed significantly between subjects, *p* < 0.05; further pairwise comparisons revealed that the peak latency of the older control group was significantly smaller than that of the youth control group (*p* < 0.05). The longer peak latency of N170 implies deeper and more detailed processing in the face recognition phase, which is consistent with the high correctness rate of the youth group in the learning phase for emotion discrimination and the testing phase for old and new discrimination. N170 may be a potential reason for the high correctness rate produced by the youth group.

ERP studies of N3 reflective valence processing have yielded inconsistent results, with some studies suggesting lower N3 wave amplitudes induced by negative emotions and others suggesting higher N3 wave amplitudes induced by negative emotions [37,38]. The N3 frontal area peak in the present study, with significant within-subject variation in face recognition, is consistent with the latter claim. Pairwise comparisons revealed that neutral faces evoked significantly smaller wave amplitudes than negative ones. There are two possible reasons for these findings. Different types of experiments and different experimental materials were chosen, and even though the same potencies were chosen for emotional face information, there were differences in emotional face picture processing. Negative emotions generally include several faces of fear, anger, surprise, and disgust, and these emotional categories also have different brain processing mechanisms, which may also lead to biased results. In the Rowanbo study, positivity induced a greater N3 wave amplitude than neutrality and negativity, with neutral faces inducing the smallest wave amplitude. For the N3 peak in the present study, the wave amplitude evoked by neutral faces was significantly smaller than that of negative faces.

Sutton first discovered in 1965 that the set of waves recorded during “cognition” of a target stimulus, including N1, P1, N2, and P3, are all endogenous components of the ERP, and later found that P3 is the most important component of cognitive function, being a positive potential recorded from the top of the head 300 ms after stimulation [39]. It has been suggested that the P300 latency reflects the speed and process of stimulus discrimination when the occasion of memory changes; that the magnitude of the wave depends on the amount of the occasion change; that the more pronounced and larger the wave of P3, the greater the likelihood of remembering the content; and that the magnitude of the P3 latency and wave amplitude are positively correlated with the magnitude of task difficulty. In the present study, the peak of the P3 parietal region differed significantly between hemispheric regions within subjects, with the central peak significantly larger than that of the left and right hemispheres. The main effect of face recognition was significant, *p* < 0.01; the main effect between subject groups was significant, with the older controls having a significantly smaller peak in the top region of P3 than the younger controls (*p* < 0.05). This indicates that the degeneration of emotional cognitive function in old age is reflected by neural mechanisms.

Fabiani et al. suggested the deterioration in emotional cognitive function in old age may be caused by the absence of memory templates for stimuli in P3a. Stimuli without memory templates are more novel and meet the conditions for evoking a stimulus toward P3a, and as the stimulus is repeatedly stimulated, the stimulus template is then formed and the P3a toward the response disappears. The brain of the elderly, especially the frontal lobe, is subjected to the effects of aging and may not easily form a template for maintaining the stimulus, and for repeated stimuli, may be more induced on P3a and less likely to be induced on P3b [40,41]. Similarly, Knight (1997) found that frontal lobe injury decreases P3a [42].

Previous studies of event-related potentials in exercise cognition have shown that physical exercise can be effective in reducing the cognitive decline typically experienced by older adults, and neurobiological assessments of cognitive function have shown that P300 indicators of event-related potentials in particular are affected by aging and that tai chi exercise may be more beneficial than walking exercise, with both exercises showing progressively shorter latencies, higher amplitudes, and shorter response times [43]. In this study, the effects of both types of exercise on the P300 showed a gradual decrease in latency, increase in amplitude, and decrease in response time. Studies have shown that components such as N170, which are triggered by specific stimulus paradigms, are associated with the cognitive processing of emotions. In the present study, the peak latency of N170 in the frontal region was significantly smaller in the elderly control group than in the young control group (*p* < 0.05); the peak of P3 in the parietal region was significantly smaller in the elderly control group than in the young control group (*p* < 0.05). These findings indicate that there is a degenerative trend in the emotional cognitive function of the elderly.

#### 4.2.2. Effects of Walking and Tai Chi Exercise on the ERP Old–New Effect of Emotional Face Memory in Older Adults

The early frontal old–new effect (or FN400), approximately 300 to 500 ms after stimulus onset, is most typical in the central frontal area, and some researchers have suggested that this component represents familiarity [44], a purely intuitive response to an old stimulus when no details are recalled; this effect is significantly greater for negative pictures than for positive pictures and for emotional words than for neutral words. It was found that unpleasant pictures induced a greater ERP old–new effect than pleasant pictures and that negative and positive words had a greater old–new effect than neutral words [45,46], and this result was confirmed by the fact that the mean ERP wave amplitude induced by negative faces in the 380–500 ms frontal area was significantly greater than that of neutral faces in the subjects of this study. Another opposing view suggests that the old–new effect in the central frontal area is related to the initiation of concepts in memory [47]. The main reason for the entanglement of these two views is that, in most experiments, familiarity and concept priming are mixed and intertwined.

The early parietal area old–new effect, around 500–700 ms, is related to the strength of the memory trace, and some studies of ERPs in young adults have reported that negative information elicits a larger parietal old–new effect than neutral information, while the prefrontal old–new effect is influenced by negative emotions or equally influenced by both positive and negative information [38]. Previous research suggested that there is a moderating effect of emotion on the effects of memory; that this moderating effect can be reflected by ERP old and new effects; and that the moderating effect of emotional ERPs varies with the method of recording, stimulus, and person relevance and emotional state [48]. Although a number of studies have shown differences in the processing of emotional stimuli between young and old people [49], the effect of emotion on the old and new effects is larger for young subjects, and the difference between emotions in modulating the memory of older and younger people is not clear. In the present study, there were significant differences in the mean ERP wave amplitude evoked in the top region from 500 to 700 ms between subjects in different groups, and the mean wave amplitude was significantly smaller in the elderly control group than in the young control group (*p* < 0.01), indicating that there is degradation of emotional face memory in the elderly.

In the present study, the mean wave amplitude of ERP evoked in the 380–500 ms and 500–700 ms top regions was greater in the elderly walking group than in the elderly control group, but the difference was not significant (*p* > 0.05). The mean wave amplitude of the elderly tai chi group was not significantly different from that of the elderly control group (*p* > 0.05). The mean wave amplitude of the elderly walking group was greater than that of the elderly tai chi group, but the difference was not significant (*p* > 0.05). These findings suggest that long-term walking and tai chi exercise have no significant effect on the old and new ERP effects related to mood in older adults.

The results of the combined behavioral and ERP analyses confirm that long-term walking and tai chi exercise have a positive effect on delaying the deterioration of emotional cognitive function in older adults. Tai chi exercise could delay the decline in aging-related emotional face recognition function to a certain extent, and walking exercise could delay the decline in aging-related emotional face memory function to a certain extent; in this regard, the effect of walking exercise was better than that of tai chi exercise. This indicates that, compared with being sedentary or engaging in occasional exercise, long-term participation in walking and tai chi exercise can delay the deterioration of emotional cognition, which is conducive to improving quality of life and promoting physical and mental health.

There are some limitations in this study. Firstly, the effects of long-term walking and tai chi exercise on emotional cognition in the elderly were investigated, and an in-depth study with a larger sample size will help to further reveal the effects and mechanisms of this phenomena. Secondly, the ERP technique has the advantage of high temporal resolution. However, the ERP technique has low spatial resolution. A combination of the neuroimaging technique with high spatial resolution, such as functional magnetic resonance imaging (fMRI), will better clarify the underlying mechanisms.

## 5. Conclusions

Behavioral and ERP component analyses indicate that there is a degradation of emotional cognitive function in the elderly. Long-term walking and tai chi exercise can delay the deterioration of emotional cognitive function in older adults to a certain extent. Both walking and tai chi exercise can delay the decline in emotional face recognition function associated with aging to a certain extent, and both exercise methods have the same effect. Walking exercise can delay the decline in aging-related emotional face memory function to a certain extent, while tai chi exercise has no significant effect. Long-term walking and tai chi exercise have no significant effects on the emotion-related ERP old- and new-age effects in older adults.

## Figures and Tables

**Table 1:** Demography of the subjects, means (SD).

	Age (Years)	Height (cm)	Weight (kg)	Years of Exercise (Years)
Young Control	22.56 ± 2.21	163.34 ± 6.82	64.52 ± 8.61	
Elderly Control	69.33 ± 5.64	161.51 ± 6.12	66.31 ± 7.31	
Elderly Walking	69.31 ± 5.41	163.19 ± 5.91	65.07 ± 4.57	9.1 ± 2.3
Elderly Tai Chi	70.63 ± 3.32	162.01 ± 5.73	65.92 ± 6.89	9.00 ± 2.1

**Table 2:** Emotional valence and arousal associated with the learning task.

		*n*	Mean	*SD*	*t*	*p*
Valence	Negative Faces	32	2.792	0.492	−10.960	<0.001 ***
	Neutral Faces	32	4.267	0.581		
Arousal Associated	Negative Faces	32	5.979	1.351	9.156	<0.001 ***
	Neutral Faces	32	3.657	0.483		

*** *p* < 0.001.

**Table 3:** Emotional valence and arousal associated with the memory task.

		*n*	Mean	*SD*	*t*	*p*
Valence	Negative Faces	84	2.836	0.501	−17.919	<0.001 ***
	Neutral Faces	84	4.206	0.490		
Arousal Associated	Negative Faces	84	6.313	1.108	17.258	<0.001 ***
	Neutral Faces	84	3.869	0.677		

*** *p* < 0.001.

## Data Availability

Data sharing not applicable.

## References

[1] Brivio P., Paladini M.S., Racagni G., Riva M.A., Calabrese F., Molteni R. (2019). From Healthy Aging to Frailty: In Search of the Underlying Mechanisms. Curr. Med. Chem..

[2] Wetherell J.L., Reynolds C.A., Gatz M., Pedersen N.L. (2002). Anxiety, Cognitive Performance, and Cognitive Decline in Normal Aging. J. Gerontol. Ser. B Psychol. Sci. Soc. Sci..

[3] Farrell T.W., Luptak M.K., Supiano K.P., Pacala J.T., de Lisser R. (2018). State of the Science: Interprofessional Approaches to Aging, Dementia, and Mental Health. J. Am. Geriatr. Soc..

[4] Verweij M., Senior T.J. (2015). Social theory and the cognitive-emotional brain. Behav. Brain Sci..

[5] Anderson A.K., Wais P.E., Gabrieli J.D.E. (2006). Emotion enhances remembrance of neutral events past. Proc. Natl. Acad. Sci. USA.

[6] Heilman K.M. (1997). The neurobiology of emotional experience. J. Neuropsychiatry Clin. Neurosci..

[7] Woodard K., Pollak S.D. (2020). Is there evidence for sensitive periods in emotional development?. Curr. Opin. Behav. Sci..

[8] Mroczek D.K. (2010). Age and Emotion in Adulthood. Curr. Dir. Psychol. Sci..

[9] Carstensen L.L., Mikels J.A., Mather M. (2006). Aging and the Intersection of Cognition, Motivation, and Emotion. Handbook of the Psychology of Aging.

[10] Charles S.T., Carstensen L.L. (2010). Social and Emotional Aging. Annu. Rev. Psychol..

[11] Stiles J. (2000). Neural plasticity and cognitive development. Dev. Neuropsychol..

[12] Berlucchi G. (2011). Brain plasticity and cognitive neurorehabilitation. Neuropsychol. Rehabil..

[13] Yeh H.-P., Stone J.A., Churchill S.M., Wheat J.S., Brymer E., Davids K. (2015). Physical, Psychological and Emotional Benefits of Green Physical Activity: An Ecological Dynamics Perspective. Sports Med..

[14] Hupin D., Roche F., Edouard P. (2015). Physical Activity and Successful Aging: Even a Little Is Good. JAMA Intern. Med..

[15] Umpierre D., Ribeiro P.A.B., Kramer C.K., Leitão C.B., Zucatti A.T.N., Azevedo M.J., Gross J.L., Ribeiro J.P., Schaan B.D. (2011). Physical Activity Advice Only or Structured Exercise Training and Association with HbA_1c_Levels in Type 2 Diabetes: A systematic review and meta-analysis. JAMA.

[16] Hamer M., Lavoie K.L., Bacon S.L. (2014). Taking up physical activity in later life and healthy ageing: The English longitudinal study of ageing. Br. J. Sports Med..

[17] Gill D.L., Hammond C.C., Reifsteck E.J., Jehu C.M., Williams R.A., Adams M.M., Lange E.H., Becofsky K., Rodriguez E., Shang Y.-T. (2013). Physical Activity and Quality of Life. J. Prev. Med. Public Health.

[18] Ruiz-Comellas A., Valmaña G.S., Peña J.M., Poch P.R., Carrera A.S., Pujol I.C., Baena I.G., Solà C., Vila C.S., Gamisans M.F. (2021). Physical activity, emotional state and socialization in the elderly: Study protocol for a clinical multicentre randomized trial. J. Int. Med Res..

[19] Kang S.-J., Kim B.-H., Lee H., Wang J. (2021). The Beneficial Effects of Cognitive Walking Program on Improving Cognitive Function and Physical Fitness in Older Adults. Healthcare.

[20] Lange-Maia B.S., Newman A.B., Cauley J.A., Boudreau R.M., Jakicic J.M., Caserotti P., Glynn N.W., Harris T.B., Kritchevsky S.B., Schwartz A.V. (2015). Sensorimotor Peripheral Nerve Function and the Longitudinal Relationship with Endurance Walking in the Health, Aging and Body Composition Study. Arch. Phys. Med. Rehabil..

[21] Lou L., Zou L., Fang Q., Wang H., Liu Y., Tian Z., Han Y. (2017). Effect of Taichi Softball on Function-Related Outcomes in Older Adults: A Randomized Control Trial. Evid. -Based Complement. Altern. Med..

[22] Ji Z., Li A., Feng T., Liu X., You Y., Meng F., Wang R., Lu J., Zhang C. (2017). The benefits of Tai Chi and brisk walking for cognitive function and fitness in older adults. PeerJ.

[23] Chang Y.K., Nien Y.H., Tsai C.L., Etnier J.L. (2010). Physical activity and cognition in older adults: The potential of Tai Chi Chuan. J. Aging Phys. Act..

[24] Di Domenico A., Palumbo R., Mammarella N., Fairfield B. (2015). Aging and emotional expressions: Is there a positivity bias during dynamic emotion recognition?. Front. Psychol..

[25] Di Loreto S., Falone S., D’Alessandro A., Santini S., Sebastiani P., Cacchio M., Amicarelli F. (2014). Regular and moderate exercise initiated in middle age prevents age-related amyloidogenesis and preserves synaptic and neuroprotective signaling in mouse brain cortex. Exp. Gerontol..

[26] Haj M.E., Fasotti L., Allain P. (2015). Destination Memory for Emotional Information in Older Adults. Exp. Aging Res..

[27] Chan A.W., Yu D.S., Choi K.C., Lee T.F.D., Sit J., Chan H.Y.-L. (2016). Tai chi qigong as a means to improve night-time sleep quality among older adults with cognitive impairment: A pilot randomized controlled trial. Clin. Interv. Aging.

[28] Ma J., Yang B., Luo R., Ding X. (2019). Development of a facial-expression database of Chinese Han, Hui and Tibetan people. Int. J. Psychol..

[29] Ally B.A., Budson A.E. (2007). The worth of pictures: Using high density event-related potentials to understand the memorial power of pictures and the dynamics of recognition memory. NeuroImage.

[30] Hatta A., Nishihira Y., Kim S.R., Kaneda T., Kida T., Kamijo K., Sasahara M., Haga S. (2005). Effects of Habitual Moderate Exercise on Response Processing and Cognitive Processing in Older Adults. Jpn. J. Physiol..

[31] Park D.C., Reuter-Lorenz P. (2009). The Adaptive Brain: Aging and Neurocognitive Scaffolding. Annu. Rev. Psychol..

[32] Dustman R., Shearer D., Emmerson R. (1993). EEG and event-related potentials in normal aging. Prog. Neurobiol..

[33] Grühn D., Smith J., Baltes P.B. (2005). No aging bias favoring memory for positive material: Evidence from a heterogeneity-homogeneity list paradigm using emotionally toned words. Psychol. Aging.

[34] Budson A.E., Todman R.W., Chong H., Adams E.H., Kensinger E., Krangel T.S., Wright C.I. (2006). False Recognition of Emotional Word Lists in Aging and Alzheimer Disease. Cogn. Behav. Neurol..

[35] Gallo D.A., Foster K.T., Johnson E.L. (2009). Elevated false recollection of emotional pictures in young and older adults. Psychol. Aging.

[36] Itier R.J. (2004). N170 or N1? Spatiotemporal Differences between Object and Face Processing Using ERPs. Cereb. Cortex.

[37] Finnigan S., Humphreys M.S., Dennis S., Geffen G. (2002). ERP 'old/new' effects: Memory strength and decisional factor(s). Neuropsychologia.

[38] Schefter M., Knorr S., Kathmann N., Werheid K. (2012). Age differences on ERP old/new effects for emotional and neutral faces. Int. J. Psychophysiol..

[39] Sutton S., Braren M., Zubin J., John E.R. (1965). Evoked-potential correlates of stimulus uncertainty. Science.

[40] Coles M., Gratton G., Fabiani M. (2000). Event-related brain potentials. Nihon Rinsho Jpn. J. Clin. Med..

[41] Fabiani M., Friedman D. (2010). Changes in brain activity patterns in aging: The novelty oddball. Psychophysiology.

[42] Woods D.L., Knight R.T. (1986). Electrophysiologic evidence of increased distractibility after dorsolateral prefrontal lesions. Neurology.

[43] Lee J., Sung J., Choi M. (2019). The factors associated with subjective cognitive decline and cognitive function among older adults. J. Adv. Nurs..

[44] Rugg M.D., Curran T. (2007). Event-related potentials and recognition memory. Trends Cogn. Sci..

[45] Inaba M., Nomura M., Ohira H. (2005). Neural evidence of effects of emotional valence on word recognition. Int. J. Psychophysiol. Off. J. Int. Organ. Psychophysiol..

[46] Weymar M., Löw A., Modess C., Engel G., Gründling M., Petersmann A., Siegmund W., Hamm A.O. (2010). Propranolol selectively blocks the enhanced parietal old/new effect during long-term recollection of unpleasant pictures: A high density ERP study. NeuroImage.

[47] Paller K.A., Voss J.L., Boehm S.G. (2007). Validating neural correlates of familiarity. Trends Cogn. Sci..

[48] Olofsson J.K., Nordin S., Sequeira H., Polich J. (2008). Affective picture processing: An integrative review of ERP findings. Biol. Psychol..

[49] Mather M., Knight M. (2005). Goal-directed memory: The role of cognitive control in older adults' emotional memory. Psychol. Aging.

